# The OncoFinder algorithm for minimizing the errors introduced by the high-throughput methods of transcriptome analysis

**DOI:** 10.3389/fmolb.2014.00008

**Published:** 2014-08-26

**Authors:** Anton A. Buzdin, Alex A. Zhavoronkov, Mikhail B. Korzinkin, Sergey A. Roumiantsev, Alexander M. Aliper, Larisa S. Venkova, Philip Y. Smirnov, Nikolay M. Borisov

**Affiliations:** ^1^Group for Genomic Regulation of Cell Signaling Systems, Shemyakin-Ovchinnikov Institute of Bioorganic Chemistry, Russian Academy of SciencesMoscow, Russia; ^2^Laboratory of Bioinformatics, D. Rogachyov Federal Research Center of Pediatric Hematology, Oncology and ImmunologyMoscow, Russia; ^3^Pathway PharmaceuticalsWan Chai, Hong Kong; ^4^Laboratory of Systems Biology, A.I. Burnasyan Federal Medical Biophysical CenterMoscow, Russia

**Keywords:** signalome, RNA-Seq, intracellular signaling pathway activation, next generation sequencing, microarray hybridization, gene expression, transcriptome profiling, correction of errors

## Abstract

The diversity of the installed sequencing and microarray equipment make it increasingly difficult to compare and analyze the gene expression datasets obtained using the different methods. Many applications requiring high-quality and low error rates cannot make use of available data using traditional analytical approaches. Recently, we proposed a new concept of signalome-wide analysis of functional changes in the intracellular pathways termed OncoFinder, a bioinformatic tool for quantitative estimation of the signaling pathway activation (SPA). We also developed methods to compare the gene expression data obtained using multiple platforms and minimizing the error rates by mapping the gene expression data onto the known and custom signaling pathways. This technique for the first time makes it possible to analyze the functional features of intracellular regulation on a mathematical basis. In this study we show that the OncoFinder method significantly reduces the errors introduced by transcriptome-wide experimental techniques. We compared the gene expression data for the same biological samples obtained by both the next generation sequencing (NGS) and microarray methods. For these different techniques we demonstrate that there is virtually no correlation between the gene expression values for all datasets analyzed (*R*^2^ < 0.1). In contrast, when the OncoFinder algorithm is applied to the data we observed clear-cut correlations between the NGS and microarray gene expression datasets. The SPA profiles obtained using NGS and microarray techniques were almost identical for the same biological samples allowing for the platform-agnostic analytical applications. We conclude that this feature of the OncoFinder enables to characterize the functional states of the transcriptomes and interactomes more accurately as before, which makes OncoFinder a method of choice for many applications including genetics, physiology, biomedicine, and molecular diagnostics.

## Introduction

The complex machinery of intracellular signaling determines the cell fate by governing all the most important processes including growth, differentiation, proliferation, migration, survival, and death. The molecular modeling of intracellular signaling pathways has been underway for more than two decades (Kholodenko et al., 1999; Hanahan and Weinberg, 2000). During that time a plethora of regulatory cascades have been discovered and cataloged (Nikitin et al., 2003; Mathivanan et al., 2006; Elkon et al., 2008; Bauer-Mehren et al., 2009; Haw and Stein, 2012; Nakaya et al., 2013). Each of these cascades contains dozens to hundreds of different types of molecules, mainly genomic DNA-encoded gene products. The information on the architecture of the signaling pathway can be used for the mathematical analysis of signal transduction process (Nikitin et al., 2003; Mathivanan et al., 2006; Elkon et al., 2008; Bauer-Mehren et al., 2009; Haw and Stein, 2012; Nakaya et al., 2013; Yizhak et al., 2013).

This information has resulted in the accumulation of the large collections of public gene expression datasets including the Gene Expression Omnibus (GEO) (http://www.ncbi.nlm.nih.gov/geo/), Stanford Microarray Database (http://smd.stanford.edu/), Cancer Genome Atlas (https://tcga-data.nci.nih.gov/), EBI ArrayExpress (http://www.ebi.ac.uk/arrayexpress/), mAdb (https://madb.nci.nih.gov/), and the many commercial repositories providing analytical services. However, until very recently, these data repositories did not include pathway-based quantitative evaluation of the functional changes between different biological samples. Additionally, there were no technical possibilities to analyze large-scale signalome signatures in high-throughput gene expression data.

Recently, we developed a set of bioinformatics algorithms and tools collectively called the OncoFinder (Buzdin et al., 2014), which encompasses the molecular signaling databases and enables performing the quantitative analysis of the signalome changes. This method digests the high throughput transcriptomic data and provides molecular signaling pathway activation (SPA) fingerprints for the individual samples.

The major distinction and novelty of the OncoFinder method is its unique algorithm that quantifies perturbations for each signaling pathway. This makes it possible to quantitatively estimate the extent of each SPA in a given sample relative to the control sample or a set of control samples (Buzdin et al., 2014).

In this study, we compared different gene expression datasets generated for the same biological samples using two different experimental techniques, *next generation sequencing* (NGS) and *microarray hybridization*. We showed that there is generally a very weak correlation between the initial NGS and microarray gene expression data. However, applying the OncoFinder method results in identifying pathway activation profiles that are highly correlated between the NGS and microarray datasets. We conclude that the OncoFinder algorithm efficiently removes errors introduced by the different experimental methodologies and yields accurate results that can be effectively used for various applications in biology, medicine and molecular diagnostics.

According to the International Aging Research Portfolio (http://www.agingportfolio.org/), over six billion dollars in government funding were spent on research projects involving microarray systems for gene expression analysis since 1993 (Zhavoronkov and Cantor, 2011) and resulted in tens of thousands of publications. And while the emerging NGS systems are providing for more-data rich and error-prone RNA sequencing methods, there is a significant installed base of the microarray systems generating new gene expression data sets. The high correlation of the pathway activation profiles of gene expression data from the same biological samples between the microarray platforms as well as NGS equipment presents the opportunity to compare the many microarray datasets generated over the past decades with the data sets obtained using the new NGS platforms.

## Results and discussion

Recently, we proposed a new concept of signalome-wide analysis of functional changes in the intracellular pathways termed OncoFinder (Buzdin et al., 2014) and developed a bioinformatic instrument for quantitative estimation of SPA. The underlying algorithm of OncoFinder converts the results of transcriptome profiling into a quantitative and qualitative signalome profile, which characterizes the states called *pathway activation strength* (*PAS*), for each signaling pathway under investigation. The information is processed based on the “low-level” transcriptomic data represented by the aggregate of the so-called *case-to-normal ratio* values, *CNR_n_*, i.e., the ratio of the expression levels of a gene *n* in the investigated sample (e.g., of an individual pathological tissue sample) to a control (e.g., average value for group of the healthy tissue samples). The major distinction of our algorithm compared to other related approaches (Yizhak et al., 2013), is that it deals with the functional annotation of the gene product and its role in the individual pathway (e.g., activator or repressor of the signal transduction through the pathway). The absolute value of PAS is characterized by the degree of functional changes in the regulation of a signaling pathway, and the positive or negative sign of PAS indicates whether it is up- or down-regulated, respectively (Buzdin et al., 2014).

This algorithm was applied to the analysis of various human tissues and cell types (Buzdin et al., 2014; Vishnyakova et al., in press) including hematologic cancers (Spirin et al., 2014) and validated (Buzdin et al., 2014) on the previously established “low-level” kinetic protein interaction model of the EGFR pathway activation (Kuzmina and Borisov, 2011). While genetics of aging and longevity are complex, the knowledge base is rapidly increasing (Moskalev et al., 2014), cancer is related to aging (Blagosklonny and Campisi, 2008) and anti-cancer agents may act as geroprotectors (Blagosklonny, 2012) and geroprotectors may provide be used in cancer prevention (Blagosklonny, 2013). The ability of the OncoFinder algorithm to perform the cross-platform comparison of the gene expression data resulted in the first proposal to use PAS in aging research for screening and ranking of the geroprotective drugs (Zhavoronkov et al., 2014).

In this study, we aimed to investigate whether the OncoFinder algorithm may be applied to minimize the error rates in gene expression data obtained using different experimental methods. We extracted the matching large-scale transcriptomic data obtained using the different experimental platforms for the same biological samples. Using the GEO repository of gene expression data (http://www.ncbi.nlm.nih.gov/geo/), we were able to extract three datasets corresponding to the simultaneous NGS and microarray profiling of the same human tissue samples. One of these datasets, GSE36244 represented human hepatocarcinomal HepG2 cells treated with benzopyrene, and the data were obtained by hybridization on the Affymetrix Human Genome U133 Plus 2.0 arrays and using the Illumina Genome Analyzer sequencing engine (van Delft et al., 2012). In the next dataset, GSE41588, published by another group of the authors (Xu et al., 2013) the same two experimental platforms were utilized for probing gene expression of the human colon cancer cell line HT-29 treated with 5-aza-deoxy-cytidine. Finally, the third dataset GSE37765 (Kim et al., 2013) included the information obtained using the Agilent 1M CNV arrays and the Illumina Genome Analyzer platform for the human female lung adenocarcinoma and for the normal lung samples. The overview of input data is presented in Table 1.

**Table 1:** **Transcriptomic data deposited in the GEO database that were used for the current study**.

**Dataset ID**	**Origin**	**Case samples vs. control samples**	**Experimental platforms**
GSE36244	HepG2 cells	Treated vs. untreated with benzopyrene	Affymetrix human genome U133 Plus 2.0 arrays and illumina genome analyzer sequencer
GSE41588	HT-29 cells	Treated vs. untreated with 5-aza-deoxy-cytidine	Affymetrix human genome U133 Plus 2.0 arrays and illumina genome analyzer sequencer
GSE37765	Lung adenocarcinoma	Tumor samples vs. matched samples of normal tissue	Agilent 1M CNV arrays and illumina genome analyzer sequencer

To apply the OncoFinder technique for signalome analysis of these datasets, we interpreted the untreated cell culture samples for the datasets GSE36244 and GSE4158, and healthy lung samples for the dataset GSE37765 to been the “normal” or control states.

**Figure 1:**
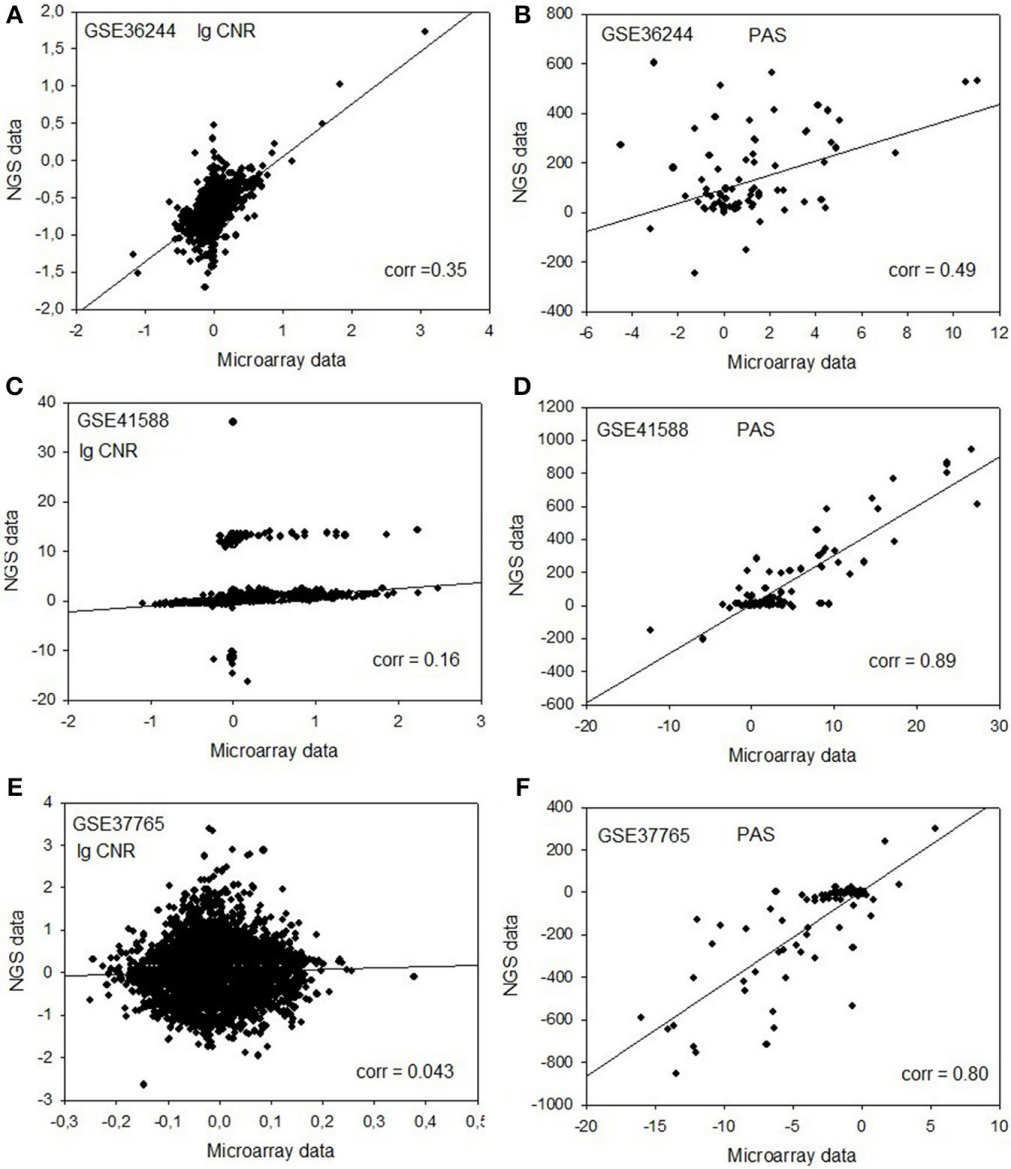
**Clouds of values obtained using the RNA next-generation sequencing vs. RNA microarray analysis methods**. Upper row **(A,B)**: cell replica 1, 24 h after BaP treatment from the HepG2 cells, dataset GSE36244 (van Delft et al., 2012). Middle row **(C,D)**: treatment with 5 μM of 5-Aza and cell replica 1 from the HT-29 cells, dataset GSE41588 (Xu et al., 2013). Lower row **(E,F)**: sample P8 from the lung adenocarcinoma dataset GSE37765 (Kim et al., 2013). Left column **(A,C,E)**: values of decimal logarithmic *CNR* for each gene. Right column **(B,D,F)**: values of *PAS*.

Next, for each dataset we compared the transcriptomic signatures obtained by the NGS and microarray hybridization platforms. All the microarray data were quantile normalized. For further normalization of the transcription data to the control samples, we calculated the case-to-normal ratio (CNR). When comparing the normalized expression logarithms between the NGS and microarray expression data, we detected virtually no correlation for all the datasets under investigation, as reflected by the low correlation coefficients generally less than 0.1 for most of the samples (Figure 1, Table 2). These results suggest that there is a huge gap between the microarray and NGS expression data for all the investigated experimental platforms.

**Table 2:** **Correlation coefficients between values obtained using the RNA microarray analysis and RNA sequencing methods for the HepG2 cells dataset GSE36244 (van Delft et al., 2012), HT-29 cells dataset GSE41588 (Xu et al., 2013), and lung adenocarcinoma dataset GSE37765 (Kim et al., 2013)**.

**Sample**		**Transcriptome level (logarithmic *CNR* for different genes)**	**Signalome level (*PAS* value for different pathways)**
GSE36244, 24 h after BaP treatment	Replica 1	0.35	0.49
	Replica 2	0.10	0.47
	Averaged over 2 samples above	0.22	0.49
GSE41588, 5 μM of 5-Aza	Replica 1	0.16	0.89
	Replica 2	0.049	0.88
	Replica 3	0.047	0.80
	Averaged over 3 samples above	0.082	0.87
GSE37765	P1	0.18	0.79
	P3	0.098	0.75
	P4	0.12	0.80
	P5	−0.029	0.21
	P8	0.043	0.80
	Averaged over 5 samples above	0.068	0.77

In contrast, for the OncoFinder-processed data and PAS we detected clear-cut correlations between the NGS and microarray gene expression datasets (Figure 1, Table 2). The correlation coefficients for PAS were significantly greater than for the CNR and varied in the interval 0.49–0.89 with a single outlier of 0.21. This finding evidences that the PAS calculation algorithm produces significantly more congruent results compared to the initial gene expression signatures between the microarray and NGS datasets.

Importantly, both NGS and microarray hybridization strategies may produce a large number of errors through the stages of RNA purification, library preparation and amplification, hybridization and sequencing, and finally mapping and annotation of the reads and reading the array (Chalaya et al., 2004; Buzdin and Lukyanov, 2007; Shugay et al., 2014). It is hard to identify the errors and to find out what type of experimental assay provides more accurate data for each individual gene. It is important to minimize the errors in the transcriptomic data and, theoretically, quantitative real-time PCR might provide a solution as a reference gene expression measure. However, existing PCR platforms do not allow for making high throughput, transcriptome-scale experiments. Our approach makes it possible to surmount this obstacle as, unlike the original data, the outgoing PAS values are highly congruent among the NGS and microarray data. This effect of the OncoFinder algorithm is most likely due to its cumulative nature. The PAS value is formed by the addition of multiple individual members each representing a gene product involved in the pathway. The concentration of each individual gene product can be measured with an error, which is seen when untreated NGS vs. array data are compared, but a combination of a large number of these concentration members into a signalome-oriented network apparently diminishes an overall error, as reflected by the good correlation records.

We conclude that this feature of PAS makes it possible to more accurately measure the changes in the functional states of the cellular/tissue transcriptome and interactome across the many microarray and NGS platforms, which makes OncoFinder a method of choice for many applications including genetics, physiology, biomedicine, and molecular diagnostics.

## Materials and methods

### Source datasets

Gene expression data used in this study were downloaded from the GEO repository of transcriptomic information (http://www.ncbi.nlm.nih.gov/geo/). The following datasets were used: GSE36244 (van Delft et al., 2012) where transcriptomes were from HepG2 cells treated with benzopyrene (four samples for the treated and four for the untreated cells); GSE37765 (Kim et al., 2013) where female normal lung and lung adenocarcinoma samples were tested (six samples for the normal and 6 for the cancer samples), and GSE41588 (Xu et al., 2013) where HT-29cells were treated with 5-aza-deoxy-cytidine (six samples for the treated and three for the untreated cells).

For the dataset GSE36244 the two transcriptome datasets were generated from the Illumina Genome Analyzer IIx sequencer and also from the Affymetrix Human Genome U133 Plus 2.0 GeneChip arrays. We took untreated HepG2 cells as the controls for further calculations. The GSE41588 data set was generated from both the Illumina sequencing platform and the Affymetrix Human Genome U133 Plus 2.0 arrays. For this dataset the untreated HT-29 cells were used as the controls. For the third dataset, GSE37765 (Kim et al., 2013), the data were obtained from the Illumina Genome Analyzer IIx sequencer and the Agilent 1M CNV microarray hybridization device. The normal lung samples were used as the controls.

The signalome knowledge base developed by SABiosciences (http://www.sabiosciences.com/pathwaycentral.php) was used to determine structures of intracellular pathways, which was used for the computational algorithm OncoFinder exactly as described previously (Buzdin et al., 2014; Spirin et al., 2014; Zhavoronkov et al., 2014).

#### Functional annotation of gene expression data

We applied the OncoFinder algorithm (Buzdin et al., 2014) for the functional annotation of the primary microarray and NGS genome-wide expression data and for the calculation of the regulatory SPA scores. The extracted raw microarray expression data were quantile normalized (Bolstad et al., 2003). Our approach to the transcriptome-wide gene expression analysis applies processing of these results with the signalome knowledge base developed by SABiosciences (http://www.sabiosciences.com/pathwaycentral.php). Our algorithm utilizes a scheme that takes into account the overall impact of each gene product in the signaling pathway but ignores its position in the pathway graph. The formula used to calculate the PAS for a given sample and a given pathway *p* is as follows:

$$PS_{p}=\sum_{n}ARR_{np}\cdot BTIF_{n}\cdot \text{lg}\left(CNR_{n}\right)$$

Here the *case-to-normal ratio*, *CNR*_n_, is the ratio of expression levels for a gene *n* in the sample under investigation to the same average value for the control group of samples. The Boolean flag of *BTIF* (*beyond tolerance interval flag*) equals zero when the *CNR* value has passed simultaneously the two criteria that demark the significantly perturbed expression level from essentially normal. The first criterion is the expression level for the sample lies within the tolerance interval, where *p* > 0.05. The second criterion is the discrete value of *ARR* (*activator/repressor role*) equals to the following fixed values: −1, when the gene/protein *n* is a repressor of pathway excitation; 1, if the gene/protein *n* is an activator of pathway excitation; 0, when the gene/protein *n* can be both an activator and a repressor of the pathway; 0.5 and −0.5, respectively, if the gene/protein *n* is rather an activator or repressor of the signaling pathway *p*, respectively. The results for the 90 pathways were obtained for each sample (listed in the Supplementary file 1). Statistical tests were done using the R software package.

### Conflict of interest statement

The authors declare that the research was conducted in the absence of any commercial or financial relationships that could be construed as a potential conflict of interest.
